# Supplementary material for: The degraded contingency test fails to detect habit induction in humans
Source: PLoS One. 2025 Oct 15;20(10):e0334087. doi: 10.1371/journal.pone.0334087 (PMC12527210; doi:10.1371/journal.pone.0334087)
Supplement: S1 File — (DOCX) [file pone.0334087.s001.docx]

**S1 Appendix. Causality Rating Wording**

Experiment 1:

"To what extent does pressing the [x] key cause coins to appear on a scale from -100 (pressing the key makes you earn much less money) to +100 (pressing the key makes you earn much more money)? You can select any value ​​in between on the scale. Click with the mouse at the location that best fits your answer."

Experiments 2 & 3

" Based on the last two minutes, to what extent does pressing the [x] key cause coins to appear on a scale from -100 (pressing the key makes you earn much less money) to +100 (pressing the key makes you earn much more money)? You can select any value ​​in between on the scale. Click with the mouse at the location that best fits your answer."

**S1 Fig. Experienced ΔP, Response Rates, and Causal Ratings**


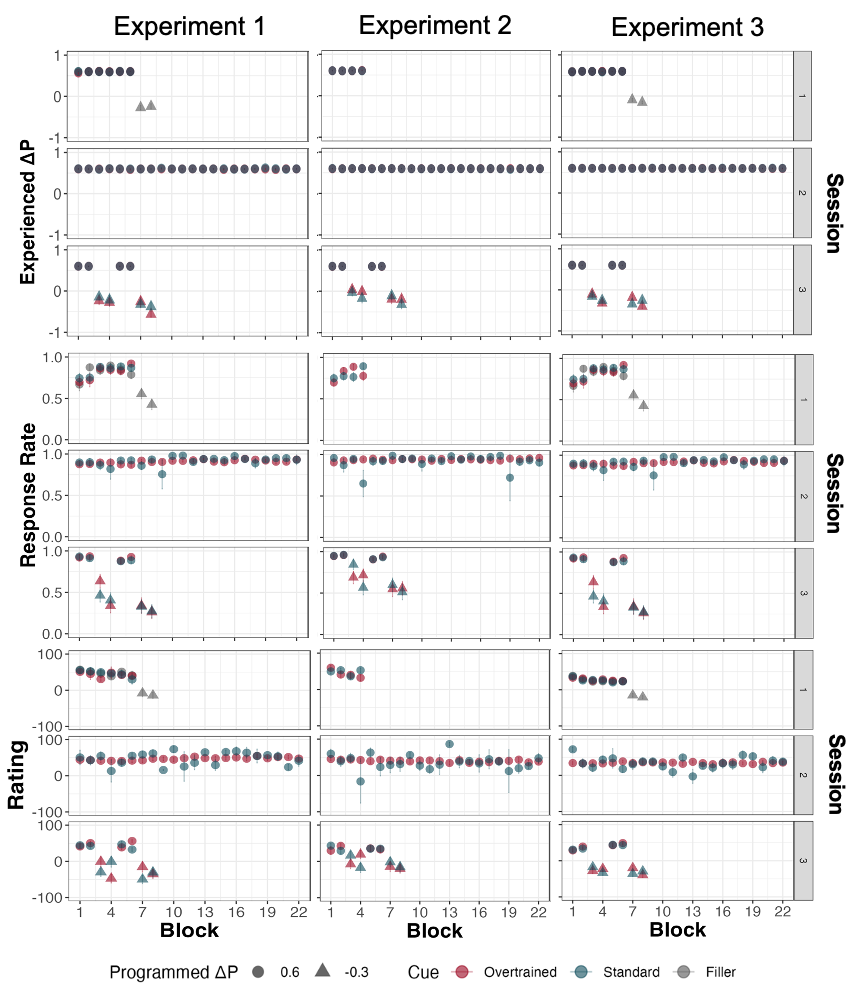


*Note.* Mean Experienced ΔP, Response Rates, and Causal Ratings across blocks and sessions for each experiment. Error bars denote the standard error.

**S2 Fig. Bayes Factor Robustness Check for each cue comparison**

**
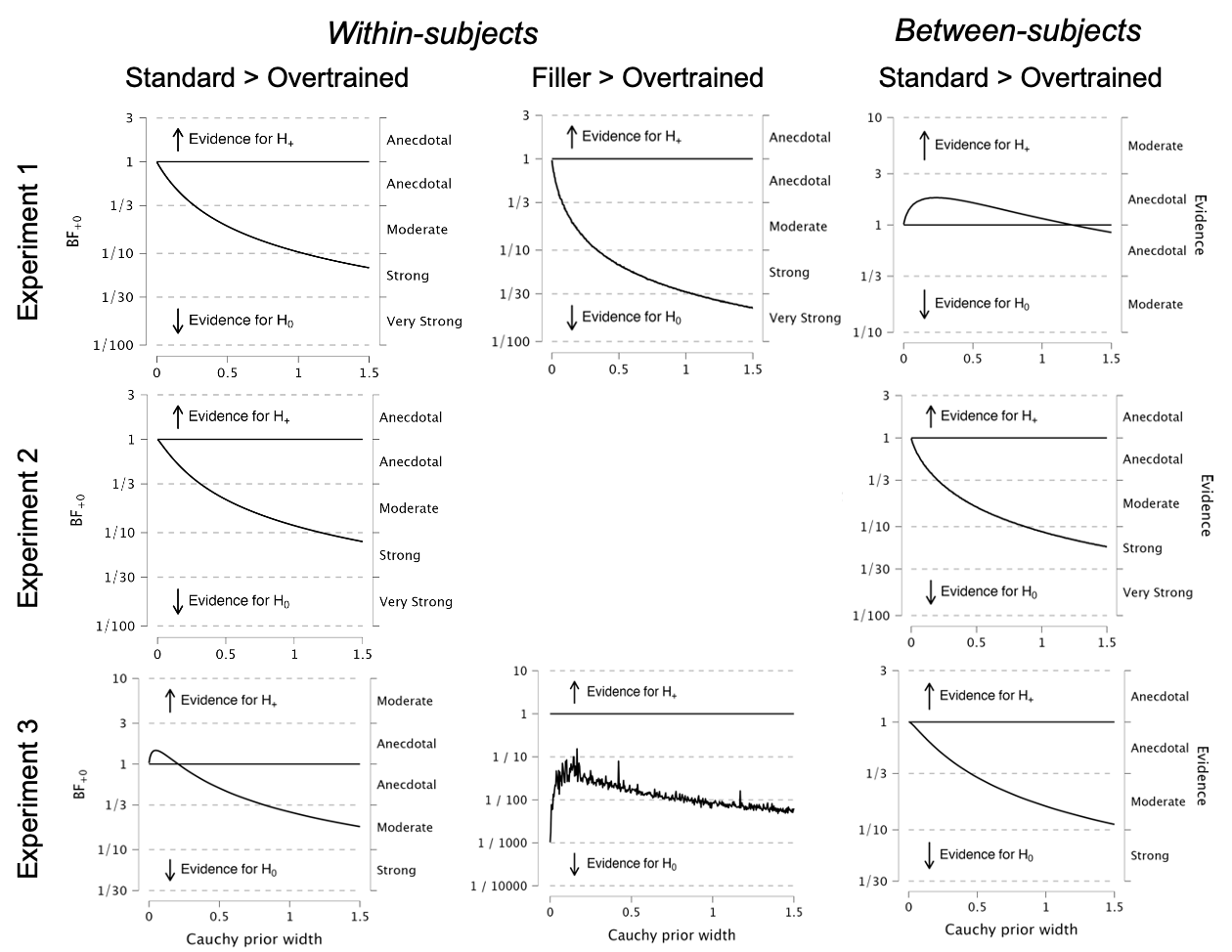
**

**S3 Fig. Correlations ratio score/causality ratings for each cue and experiment under negative contingencies (within-subject analyses).**


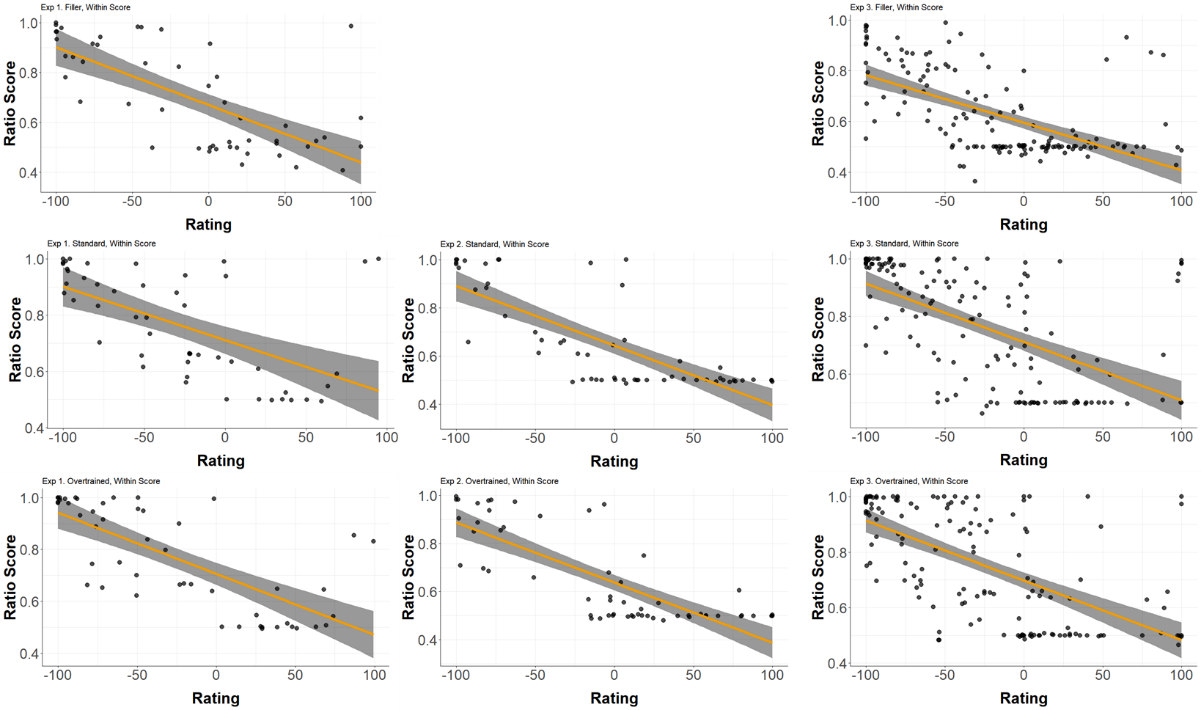


*Note*. Experiments are shown in different columns and cues in different rows.

**S1 Table. Correlations ratio score/causality ratings for each cue and experiment under negative contingencies (within-subject analyses).**

|  |  | r | Df | 95% CI | | p |
| --- | --- | --- | --- | --- | --- | --- |
|  |  |  |  | LL | UL |  |
| Exp.1 | Filler  Standard  Overtrained | -0.699  -0.577  -0.725 | 48  48  48 | -0.818  -0.737  -0.834 | -0.522  -0.357  -0.559 | <.001  <.001  <.001 |
| Exp.2 | Standard  Overtrained | -0.766  -0.786 | 55  55 | -0.856  -0.868 | -0.632  -0.660 | <.001  <.001 |
| Exp.3 | Filler  Standard  Overtrained | -0.582  -0.569  -0.607 | 145  145  145 | -0.680  -0.669  -0.700 | -0.464  -0.448  -0.494 | <.001  <.001  <.001 |

**S4 Fig. Correlations ratio score/causality ratings for each cue and experiment under negative contingencies (between-subject analyses).**


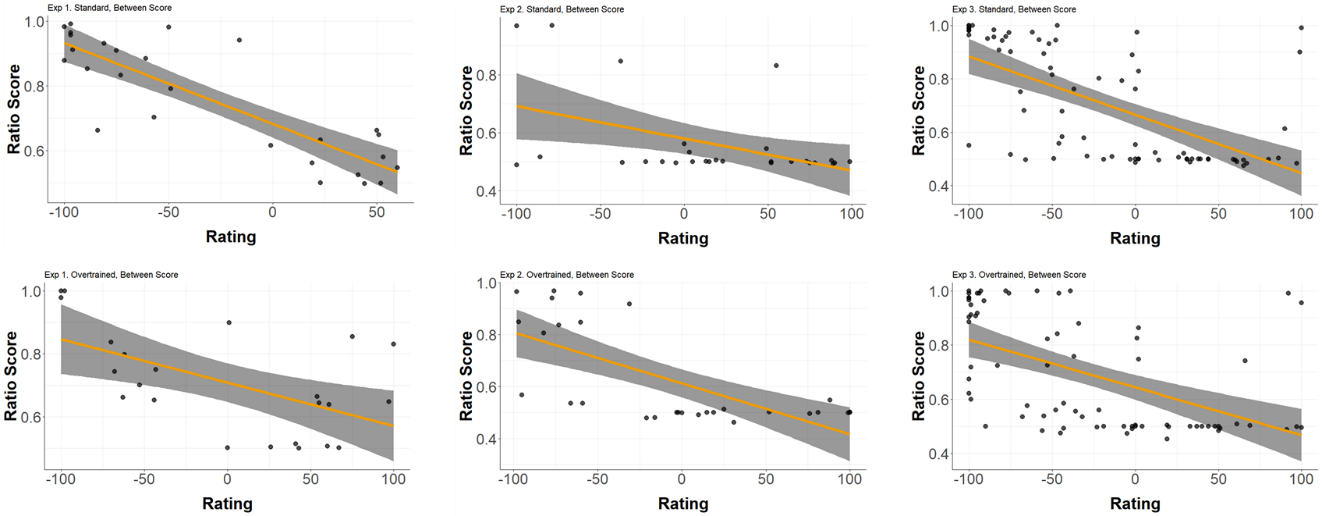


*Note*. Experiments are shown in different columns and cues in different rows.

**S2 Table. Correlations ratio score/causality ratings for each cue and experiment under negative contingencies (between-subject analyses).**

|  |  | r | Df | 95% CI | | p |
| --- | --- | --- | --- | --- | --- | --- |
|  |  |  |  | LL | UL |  |
| Exp.1 | Standard  Overtrained | -0.852  -0.558 | 25  21 | -0.931  -0.788 | -0.699  -0.189 | <.001  .005 |
| Exp.2 | Standard  Overtrained | -0.456  -0.682 | 26  27 | -0.708  -0.839 | -0.100  -0.422 | .014  <.001 |
| Exp.3 | Standard  Overtrained | -0.621  -0.521 | 72  71 | -0.744  -0.671 | -0.457  -0.331 | <.001  <.001 |
