# Supplementary material for: The degraded contingency test fails to detect habit induction in humans
Source: PLoS One. 2025 Oct 15;20(10):e0334087. doi: 10.1371/journal.pone.0334087 (PMC12527210; doi:10.1371/journal.pone.0334087)
Supplement: Attachment — Submitted filename: Response to Reviewers.docx [file pone.0334087.s003.docx]

**Journal Requirements:**

When submitting your revision, we need you to address these additional requirements.

1. Please ensure that your manuscript meets PLOS ONE's style requirements, including those for file naming. The PLOS ONE style templates can be found at

<https://journals.plos.org/plosone/s/file?id=wjVg/PLOSOne_formatting_sample_main_body.pdf> and [https://journals.plos.org/plosone/s/file?id=ba62/PLOSOne_formatting_sample_title_authors_affiliations.pdf](https://track.editorialmanager.com/CL0/https:%2F%2Fjournals.plos.org%2Fplosone%2Fs%2Ffile%3Fid=ba62%2FPLOSOne_formatting_sample_title_authors_affiliations.pdf/1/010f0197c15645b9-33f8cca3-ff1b-4322-9c97-3420b8d107b0-000000/rVkGxwADfVA0zd7pbUUo_NvdWnIsP8fKQNhcVEg8EKw=217)

2. Thank you for stating in your Funding Statement:

[This work was supported by grant PID2021-126767NB-I00 (S.M, D.L.), Ministry of Science, Innovation and Universities (MICIU); and grant PRE2022-103151 (P.M.L.), MICIU/ESF.].

Please provide an amended statement that declares *all* the funding or sources of support (whether external or internal to your organization) received during this study, as detailed online in our guide for authors at <http://journals.plos.org/plosone/s/submit-now>.  Please also include the statement “There was no additional external funding received for this study.” in your updated Funding Statement.

Please include your amended Funding Statement within your cover letter. We will change the online submission form on your behalf.

3. Thank you for stating the following in your manuscript:

[This work was supported by grant PID2021-126767NB-I00 (S.M, D.L.), Ministry of Science, Innovation and Universities (MICIU); and grant PRE2022-103151 (P.M.L.), MICIU/ESF.]

We note that you have provided funding information that is currently declared in your Funding Statement. However, funding information should not appear in the Acknowledgments section or other areas of your manuscript. We will only publish funding information present in the Funding Statement section of the online submission form.

Please remove any funding-related text from the manuscript and let us know how you would like to update your Funding Statement. Currently, your Funding Statement reads as follows:

[This work was supported by grant PID2021-126767NB-I00 (S.M, D.L.), Ministry of Science, Innovation and Universities (MICIU); and grant PRE2022-103151 (P.M.L.), MICIU/ESF.]

Please include your amended statements within your cover letter; we will change the online submission form on your behalf.

4. Please amend the manuscript submission data (via Edit Submission) to include author Sara Molinero.

5. Please amend your authorship list in your manuscript file to include author Sara Molinero Infante.

6. Please include captions for your Supporting Information files at the end of your manuscript, and update any in-text citations to match accordingly. Please see our Supporting Information guidelines for more information: [http://journals.plos.org/plosone/s/supporting-information](http://track.editorialmanager.com/CL0/http:%2F%2Fjournals.plos.org%2Fplosone%2Fs%2Fsupporting-information/1/010f0197c15645b9-33f8cca3-ff1b-4322-9c97-3420b8d107b0-000000/Ctoz2VFTzfVOjIPrOCh0_bXkuGzuPmhioeXtS4wxZOc=217).

7. Please review your reference list to ensure that it is complete and correct. If you have cited papers that have been retracted, please include the rationale for doing so in the manuscript text, or remove these references and replace them with relevant current references. Any changes to the reference list should be mentioned in the rebuttal letter that accompanies your revised manuscript. If you need to cite a retracted article, indicate the article’s retracted status in the References list and also include a citation and full reference for the retraction notice.

**Response**: We have done all the requested changes.

-o-O-o-

**Responses to Reviewers' comments**

**Reviewer #1**

**R1.1:** *The authors interpret the null effect of overtraining as evidence that participants remained goal-directed across conditions. However, from Figure 2, it appears that participants show fairly habitual behavior even after short training - particularly in Experiment 2, where performance under degraded contingencies seems completely habitual (ratio score around 0.5). This raises the possibility that both standard and overtrained cues in the task elicited habitual behavior. The null effect may actually reflect generalization of the learning/performance/habit to the standard cue (which seems reasonable in the current task in my opinion).*

*Additionally, another observation that may challenge the authors’ interpretation (and could benefit from further reflection in the paper) is the apparent bimodal distribution in response tendencies. That is, some participants appear to become habitual while others remain sensitive to contingency degradation, regardless of training duration.*

**Response:** We would like to thank the reviewer (both, reviewer 1 & 2) for raising this concern, allowing us to address this issue. Certainly, observing Experiment 2 data in Fig 2, participants seem to be quite habitual, with values close to 0.5 in ratio score even in the less trained condition (standard cue). However, we suspect that this pattern of data reflects difficulties in some participants who didn´t notice that a change in contingency occurred; if so, perseverant responses were goal-directed because for these participants the response was still a good way to get the reward. To explore this possibility, we calculated the correlation between ratio score and causality ratings during habit test (negative contingency blocks). We obtained strong and significant negative correlations between these two variables (see S3-S4 Fig, S1-S2 Table in Supplemental Material). These correlations strongly suggest that those participants who indicated a higher perception of causality, that is, a positive response-outcome contingency, are those who changed their responses the least when moving from training to test. Therefore, the seemingly “habitual” behavior observed in our results, and pointed out by the reviewer, were most likely goal-directed responses. These new results are discussed in the General Discussion.

Regarding the bimodal distribution, this type of distribution has been reported before in operant training protocols using a devaluation procedure (Gera et al., 2023; Pool et al, 2022). However, in a recent study from our lab that we are about to publish (Vázquez-Millán et al., 2025) we showed that this bimodal distribution reflects two groups of people who differ, not in whether the behavior is habitual or goal-directed, but in whether they have understood the devaluation correctly or not. In our contingency degradation protocol, and based on the new explorative analysis, the same explanation can be applied: we would have participants who have detected that the contingency is negative, and participants who have not.

**Minor points:** *1. The use of the word “unaffected” in the abstract may be too strong. A moderate reduction in behavior in degraded blocks may still reflect habitual behavior to some degree. Consider softening this claim to better reflect the nuance of the data.*

**Response:** Ok, corrected.

*2. The last paragraph in the Introduction speaks about the (series of) experiments in a way that feels they have been introduced before (while they were not). Consider rephrasing accordingly.*

**Response:** Ok, corrected.

*3. Page 5: More details on the power analysis for Experiment 1 would be helpful.*

**Response:** The effect that we were looking for has not been reported yet in the literature, that means that we couldn´t calculate the power analysis based on prior data, instead, we followed the recommendation of Brysbaert (2019). Now this is better explained in the manuscript: “Because the effect that we were looking for has not been reported previously, we did not have a previous effect size to estimate the appropriate sample size. Thus, we followed the calculations of Brysbaert’s (2019) study, which indicates how many participants are needed to have properly powered experiments, in absence of previous similar effect. This author signaled that d = .4 is a good estimate of the smallest effect size of interest in psychology research and that over 50 participants are needed for a simple comparison of two within-participants conditions (in our case, standard vs overtraining) if we want to run a study with 80% power”

*4. Table 1: The structure is confusing. The rows appear as labeled with cue types (one the left), which they are not. Consider revising the format or separating the cue labels clearly (e.g., as a separate legend or in the caption). Additionally, it says: “positive/negative or habit test” but “negative” is the habit test in this paradigm. Please revise this for clarity.*

**Response**: Noted. We have changed Table 1 to describe more clearly the design and distribution of blocks. We also added comments in the corresponding table note and the manuscript (see design and procedure in Experiment 1, method section) to state earlier that negative contingency blocks were used as habit tests.

*5. Page 8: “This was confirmed by a correlation analysis between programmed delta P and experienced delta P, r = .867, p < .001. Causality ratings further confirmed that participants were aware of contingency shifts, correctly identifying changes from positive to negative contingencies.” It is not clear if this refers to two separate analyses. Please clarify with separate reporting if so.*

**Response:** Ok, this is now clearer (there were not two separate analysis). We have also added information regarding the new correlations (p. 13).

*6. Page 9: “participants who completed only the first habit test...” is a bit misleading. As I understand it, all participants completed both tests; this is just a comparison of the first test block between groups. Please clarify.*

**Response:** That is correct, we rephrased the sentence (now in p. 14).

*7. Page 16: The claim that more extensive training would not have changed the result rests on weak evidence. The authors cite asymptotic response rates and prior studies, but:
- Reaching asymptote doesn’t preclude additional habit formation.
- Other work (e.g., Lally et al., 2010) shows habits may take a long time to form.
- Luque et al. (2020) used a different paradigm and test, so its relevance here is limited.
That said, this is the authors' interpretation, and I do not think it necessarily needs to be removed or changes. However, I do encourage them to reconsider this point.*

**Response:** Ok, we softened our claim and stated that it is possible that more training could have led to different results (see General Discusion, p. 26-27).

*8. In discussion, when discussing more effective methods of revealing habit (e.g., time pressure, stress), the authors could mention Gera et al. (2024), who recently demonstrated habit formation over extended training using a novel smartphone-based task. This would enrich the discussion of methodological innovation and bridge lab-to-real-world settings.
9. When discussing the challenges associated with outcome devaluation procedures, overtraining effects, and identifying effective methods for inducing habits, it would be worthwhile to mention recent alternative approaches, such as Gera et al. (2024) who demonstrated habit formation (devaluation insensitivity) as a function of training duration using a smartphone app paradigm that allows for more extended training.*

**Response:** Agreed. The work of Gera et al. (2024) is now mentioned in the General Discussion (p. 28).

*Other Thoughts:
1. I wonder whether excluding the first few seconds of the test blocks (where participants might learn about the contingency change) affects the results.*

**Response:** This is an interesting suggestion, however, in our opinion it would be difficult to determine how many seconds to eliminate from the analysis, especially because the time spent before realizing that a change in contingency occurred might be quite different from one participant to another. Given our results, our guess is that it would be harder to identify habitual behavior if we analyze only a portion of the (already short) habit test. We believe that we shouldn’t deviate from the analysis that are preregistered, which are in line with previous related work (Vagui et al., 2019), allowing for comparisons.

*2. The task structure/components likely prompts constant vigilance to contingency, potentially undermining habit expression.*

**Response:** The reviewer is right. The (necessary) inclusion of causality ratings and memory tests at the end of each block might have encouraged participants to be focused on the task. But this issue might be hard to avoid when we are trying to experimentally study habit induction. However, please note that we tried to keep a stable environment by maintaining the same positive contingency for all blocks of training, facilitating the development of habitual responses (that is not the case for related work as Vagui et al. 2019). It is possible that this constant vigilance is one factor that makes it necessary to somehow hinder the functioning of the goal-directed system to observe valid evidence of habits (Hardwick et al., 2019; Luque et al., 2020—this idea was discussed in the General Discussion, p. 27).

**Reviewer #2**:

*If I’ve misunderstood any aspect or you disagree with a point, a brief clarification is more than sufficient; there is no obligation to revise the manuscript based on these comments.*

*Major*
**R2.1:** *My main points relate to the interpretation of the results as showing no habit induction.
The authors convincingly demonstrate that there is no difference in outcome degradation sensitivity between the overtrained and standard conditions. However, the conclusion that this represents a failure to induce habits is open to debate. Looking at the data presented (particularly in Figure 2), a notable proportion of participants in both conditions exhibit response ratios near 0.5, which is indicative of habitual responding. This pattern is especially apparent in the between-subject comparisons, which arguably provide a cleaner measure. This raises an important alternative interpretation: rather than a failure to induce habits via overtraining, it's possible that the "standard" training protocol—conducted over three days—was already sufficient to induce robust habits in many participants. If this were the case, it would lead to a floor effect, where the overtraining has no additional effect to be measured. I would encourage the authors to discuss this possibility more thoroughly. Could the conclusion be reframed, not as a failure of the test, but as evidence that even moderate training can be sufficient to establish habitual control under these conditions?*

**Response:** Thanks for this observation. Given the simplicity of the task, it would be possible that our short condition was long enough to develop habitual responses—even though previous literature indicates that we need quite more time (eg. Lally et al., 2010). However, the new explorative analysis that we performed, now included in supplemental material (see S3-S4 Fig and S1-S2 Table), suggests a different explanation, that is, that some participants weren´t aware of the change in contingency, maintaining the same response that worked until then (please, see also first response to Reviewer 1, and General Discussion p. 22).

**R2.2:**  *The manuscript frames the findings primarily as a shortcoming of the degraded contingency test itself. This is one possibility, but I would encourage the authors to more thoroughly consider an alternative framing. While the results demonstrate failures to demonstrate habit induction with overtraining effect, this may also be a consequence of the specific experimental parameters chosen (e.g. Schreiner et al 2019). As subtle changes in experimental design can have significant effects on the expression of habitual behavior, a more cautious interpretation may be warranted. It would therefore strengthen the paper to more explicitly balance the discussion between the test being the issue versus the specific training conditions being the reason for the observed null effect. The authors mention several elements of the study design that may contribute, e.g. that habits were tested as withholding an overtrained response (which may not become habitual) and stable vs changing contingency relationships and compare this with other human studies. But it’s less clear how the current study compares with the rodent literature where overtraining effects on contingency degradation tasks are reliably found. How do the key parameters of the current study (stable vs. varying contingency relationships; specific ΔP values for the degradation phase) compare to those classic animal studies? This could help contextualize the current results and strengthen the argument regarding the critical importance of specific experimental parameters in observing habit formation.*

**Response:** It is always a challenge trying to replicate animal findings in human, especially because we cannot use exactly the same protocols that work with animals. Usually, animals have to learn new responses that are not part of their repertoire, their motivation for obtaining the rewards is maintained high through the experiment very easily (i.e., they are hungry!), the experimental environment is more stressful for them…etc. For the current study, we based our design on other human study such as Vagui et al., (2019). We explain in the manuscript in what aspects we deviated from this work. In our opinion, there is too much distance between these studies and animal experiments to include a detailed comparison between them—that would be of little use for the reader in our humble opinion. Nevertheless, at least we tried to keep a constant positive contingency relationship between events to facilitate the development of persistent responses, sharing that aspect with classical animal studies which reported overtraining effects (Dickinson et al., 1995, 1998). Thus, at least in theory, changes from positive to negative contingencies should have affected little trained responses but no overtrained responses, or at least not at the same level. We believe that the manuscript sufficiently discussed the difficulties for translating animal results to humans, pointing out that new protocols are still emerging and suggesting that we might not know yet the specific parameters to facilitate habit induction. Nevertheless, the new version of the manuscript states clearly that other parameters in our protocol might have ended up with a different result (see General Discussion section p. 27 and 29).

Minor

Methods:
• *The response ratio is (appropriately) used to obtain a relative measure of goal-directedness. However, since it would be very insightful to see (individual differences in) the absolute number of responses participants made. Would it be possible to include a (supplementary) plot showing the absolute number of responses for each cue type over test blocks? This would provides more insight into the behavior of participants to readers.*

**Response:** Done, see please S3 and S4 Fig in supplemental material.

• *The manuscript includes both within- and between-subject comparisons, but the rationale and description are not clearly articulated in the Methods section of Study 1. I found it difficult to understand at first.*

**Response:** We tried to describe more clearly our decision for implementing an exploratory between-subject analysis (see Method in Experiment 1).

• *The speed of data collection for Experiments 1 and 2 is remarkable, with each study seemingly completed within three days. This implies a very high throughput of participants. A brief sentence in the Methods explaining the logistics (e.g., use of multiple testing stations, an online platform) would be helpful and would mitigate potential reader questions about feasibility.*

**Response:** Our lab at full capacity permits testing 10 participants per hour, normally we announce 7 timeslots per day, which means that we can test up to 70 students in an overtrained condition in 3 consecutive days. This is clarified in the manuscript (see Method, Experiment 1, p 8).

Experiment 1:
• *When reporting the power analysis, could the authors specify the target effect size and the alpha level that determined a sample size of 50 participants would be sufficient?*

**Response:** Thanks for pointing this out. We added a few more details about the power analysis in the new version of the manuscript, please see method section for Experiment 1 (p 7), and also, our response to reviewer 1 (minor points, number 3).

• *The term "habit test" seems to appear for the first time in a table. It would improve clarity to introduce and define this term in the main text when the procedure is first described.*

**Response:** The new version of the manuscript states earlier that negative contingency blocks were used as habit tests (see method section in Experiment 1 and response to reviewer 1: minor points, number 4).

Experiment 2:
• *Could the authors please provide the rationale for including 57 participants in this experiment?*

**Response:** Since the effect of Exp 1 was null, we did not see fit to use it for a power analysis. Instead, we followed the same rationale as in Experiment 1. We clarified this point in the manuscript (see method section, Experiment 2).

Experiment 3:
• *The justification for Experiment 3 ("an exploratory analysis suggested a trend") is somewhat vague. It would help the reader if the authors could elaborate on the nature of this trend and the specific hypothesis that motivated this follow-up study.*

**Response:** The new version of the manuscript describes more clearly what motivated us to run a third experiment (p. 19).

• *In the Results section, evidence for overtrained vs standard cue was interpreted as strong evidence, while the BF01 was only 2.63. Seems like a small mistake.*

**Response:** Ok, thanks for pointing out this mistake. BF01 = 2.63 can be interpreted as anecdotal evidence. This error has been corrected.
